# Correcting angular distortions in Bragg coherent X-ray diffraction imaging

**DOI:** 10.1107/S1600577524006507

**Published:** 2024-08-08

**Authors:** Huaiyu Chen, Dmitry Dzhigaev, Alexander Björling, Fabian Westermeier, Mikhail Lyubomirskiy, Michael Stuckelberger, Jesper Wallentin

**Affiliations:** a Synchrotron Radiation Research and NanoLund, Department of Physics Lund University 22100Lund Sweden; b MAX IV Laboratory Lund University 22100Lund Sweden; c Deutsches Elektronen-Synchrotron DESY 22607 Hamburg Germany; Paul Scherrer Institut, Switzerland

**Keywords:** angular uncertainty, angular distortions, Bragg coherent X-ray diffraction imaging, BCDI, phase-retrieved objects, nano-scale particles, angular corrections

## Abstract

Bragg coherent X-ray diffraction imaging (BCDI) has emerged as a powerful technique for strain imaging and morphology reconstruction of nanometre-scale crystals. However, BCDI often suffers from angular distortions that appear during data acquisition, caused by radiation pressure, heating or imperfect scanning stages. This limits the applicability of BCDI, in particular for small crystals and high-flux X-ray beams. Here, we present a pre-processing algorithm that recovers the 3D datasets from the BCDI dataset measured under the impact of large angular distortions. We systematically investigate the performance of this method for different levels of distortion and find that the algorithm recovers the correct angles for distortions up to 16.4× (1640%) the angular step size dθ = 0.004°. We also show that the angles in a continuous scan can be recovered with high accuracy. As expected, the correction provides marked improvements in the subsequent phase retrieval.

## Abstract 2

An algorithm has been developed that effectively corrects and tracks angular distortions, enabling BCDI to work much more robustly and accurately in a wider range of challenging experimental scenarios.

## 1. Introduction

Bragg coherent X-ray diffraction imaging (BCDI) has evolved as a powerful and promising X-ray microscopy technique for the study of crystalline nanoparticles (Pfeifer *et al.*, 2006 ▸; Robinson & Harder, 2009 ▸; Miao *et al.*, 2015 ▸). BCDI exploits the penetration depth of X-rays and their high sensitivity to distortions of the crystalline lattice. The structural information of single particles is encoded in the 3D scattering intensities around Bragg reflections, and by phase retrieval (Fienup, 1978 ▸) of such 3D diffraction datasets, BCDI can provide the local 3D morphology and strain of a crystalline nanoparticle with high spatial resolution. The new fourth-generation synchrotron radiation sources that produce highly brilliant X-ray beams make it possible to extend BCDI to a larger range of applications (Li *et al.*, 2022 ▸) as well as smaller nanoparticles (Björling *et al.*, 2019 ▸).

**Figure 1:**
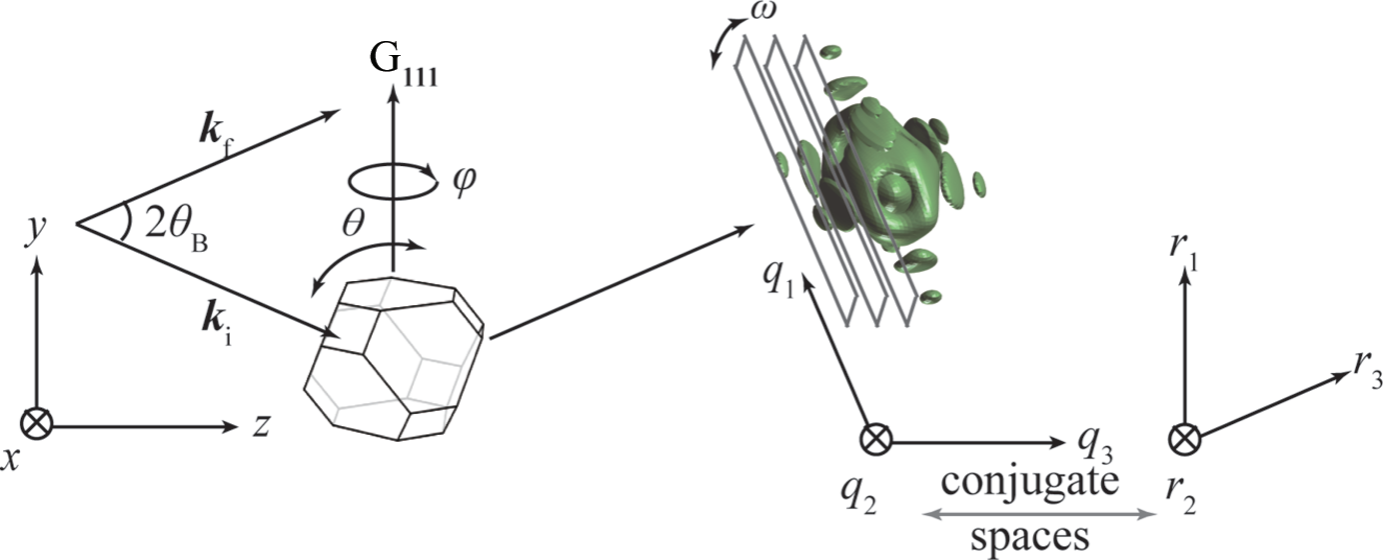
Scheme of BCDI experiment. A coherent X-ray beam with the incident wavevector **k**_*i*_ and exit wavevector **k**_*f*_ is scattered by a nanoparticle with a truncated octahedron envelope. The angle between **k**_*i*_ and **k**_*f*_ is twice the Bragg angle θ_B_ of the lattice point *G*_111_. The squares across the Bragg peak represent the detector window, whose plane (*q*_1_ × *q*_2_) is perpendicular to the exit wavevector **k**_*f*_. By rotating the sample in a small range along the rocking curve direction θ, the 3D diffraction volume in reciprocal space can be sampled by a stack of 2D slices recorded by the detector. The *q*_3_ direction in reciprocal space corresponds to the rocking curve direction θ. The conjugate space of (*q*_1_, *q*_2_, *q*_3_) is (*r*_1_, *r*_2_, *r*_3_).

In a BCDI experiment as shown in Fig. 1 ▸, the Bragg condition is met when the wavevector transfer |**Q**| = **k**_*f*_ − **k**_*i*_ (**k**_*i*_ and **k**_*f*_ are the incident and exit wavevectors, respectively) coincides with one of the reciprocal lattice points **G**_*hkl*_, with indexes (*hkl*). A detector placed at the selected Bragg angle θ_*hkl*_ at a sample-to-detector distance *r* would then capture the Bragg diffraction. The entire 3D Bragg diffraction can be recorded by rotating the sample along θ (around the *x* axis) in small steps in a so-called rocking curve. The range of the rocking curve, Δθ, is generally less than 1°, and the 2D slices can be considered as effectively parallel in the *q*_3_ direction as shown in Fig. 1 ▸. The relation between the coordinates in reciprocal space (*q*_1_, *q*_2_, *q*_3_) and those in real space (*r*_1_, *r*_2_, *r*_3_), as well as the Cartesian coordinates (*x*, *y*, *z*), are described by Berenguer *et al.* (2013 ▸).

One of the main challenges for BCDI is the angular uncertainty that can occur during the BCDI measurement procedure. Unpredictable particle rotations can be induced by an imprecise experimental platform, in particular when using complex *in situ* experimental setups, as well as movements due to radiation pressure, charging or heat (Kim *et al.*, 2016 ▸; Liang *et al.*, 2018 ▸; Hruszkewycz *et al.*, 2018 ▸). These issues are more pronounced for small nanoparticles and in more realistic sample systems where particles are weakly bound to the substrate (Björling *et al.*, 2019 ▸), but also when using nano-focused and high-flux X-ray beams. These distortions can be quite obvious in severe cases and sometimes prevent phase retrieval or result in lower-quality reconstructions. Fig. 1 ▸ illustrates the three rotations which can suffer from angular uncertainty. The first is along the rocking curve angle θ. It has previously been demonstrated in simulations that even a slight angular uncertainty along θ, much smaller than the step size, can introduce artifacts in the 3D reconstruction after the phase retrieval (Calvo-Almazán *et al.*, 2019 ▸). Such small uncertainties would be hard to recognize in an experiment. The particle roll angle ω can also be problematic, but it is relatively easy to recognize since it leads to shifts in the detector plane. Finally, the BCDI geometry makes it much less sensitive to uncertainties in the azimuthal angle φ (Björling *et al.*, 2020 ▸).

There have been some published methods working on mitigating the influence caused by angular uncertainty and improving the robustness of BCDI. Calvo-Almazan *et al.* (2019 ▸) modified the phase-retrieval step to jointly retrieve the angle and the object. The method could reconstruct the complex electron distribution ρ of the sample and estimate the exact angular position via the calculation of a well defined error metric gradient during the phasing process. According to the simulations, their method performed well for modest distortions of up to 0.4× (40%) the step size dθ from nominal rocking curve angles. At the other end of the spectrum, Björling *et al.* (2020 ▸) demonstrated a method to recover the angles from a fully unknown and uncontrolled particle rotation. Their method builds on the Expand–Maximum–Compress (EMC) algorithm (Loh & Elser, 2009 ▸; Loh *et al.*, 2010 ▸; Ekeberg *et al.*, 2015 ▸) that is generally applied in X-ray free-electron laser (XFEL) imaging experiments, and it is used as a pre-processing step independent of the phase retrieval. In this case, the particle rotation was fully driven by the beam, which is a rather extreme scenario.

Here, we investigate how the EMC based method (Björling *et al.*, 2020 ▸) can be used in a scenario with both intentional and non-intentional rotations. We only consider distortions in the θ direction, which is the most challenging direction to recognize and correct for, in order to reduce computational time. The simulations performed show that this pre-processing method performs well for deviations of up to 16.4× (1640%) the step size dθ = 0.004°. Our method builds on the theoretical framework presented by Björling *et al.* (2020 ▸), utilizing the oversampling present in collected diffraction data to correct the influence of angular uncertainty. We use phase retrieval to confirm that the angular correction results in improved reconstructions.

## 2. Details of the algorithm and simulation

In order to test the performance, we simulated BCDI measurements of 250 nm-diameter truncated-octahedral gold nanoparticles (approximately 170 nm in length in the *r*_3_ direction) at an X-ray energy of 10 keV. The particle was combined with an artificial internal phase varying between −0.25 radians and 0.25 radians. The whole 3D diffraction intensity volume around the reciprocal lattice point *G*_111_ was sampled in the angular range Δθ = ±0.4° in *N*_*k*_ = 201 angular steps (*i.e.* with a nominal dθ = 0.004°). The resulting 2D diffraction patterns were recorded by a 2D array of 256 × 256 pixels with a 55 µm pixel size, at a sample-to-detector distance of 0.5 m. A random angular perturbation δθ with the average distortion defined as Γ_θ_ = mean(δθ)/dθ was generated and added to the preset angular position θ to obtain the distorted trajectory. The generated orientation trajectory was then used as input for the software *Ptypy* (Enders & Thibault, 2016 ▸) to simulate the Bragg diffraction dataset.

The algorithm is based on the work proposed by Björling *et al.* (2020 ▸), in which a corrected model *W* is iteratively updated based on searching the maximum value of likelihood with respect to the measured frames *K* and their position in θ. The logarithm form of the likelihood *R*_*jk*_ with respect to the frames *K* (index *k*) and the angular position θ (bin *j*) is given by the probability mass function of the Poisson distribution,
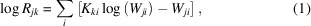
where index *i* runs over the detector pixels and will be omitted in the following. Analogous to the approach mentioned by Björling *et al.* (2020 ▸) and Ayyer *et al.* (2016 ▸), an annealing parameter β is used to avoid local optima. Then, the normalized probability matrix *P*_*jk*_ which indicates the *k*th experimental frame *K*_*k*_ corresponding to θ bin *j* can be calculated as
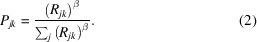
Note that *P*_*jk*_ is a probability distribution, not just the most probable angle. The final calculated *P*_*jk*_ can be used as the orientation trajectory θ(*k*) to show where the single measured diffraction pattern *K*_*k*_ belongs in rocking angle θ. A continuity bias *n*_σ_ is imposed onto *P*_*jk*_ before normalization as mentioned by Björling *et al.* (2020 ▸) to ensure the orientation trajectory θ(*k*) is single-valued. According to equation (1), the maximum value occurs when 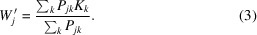
Unlike the case in an XFEL imaging experiment in transmission, where all the measured slices intersect the origin in reciprocal space, the frames in a BCDI measurement are approximately parallel to each other. The field of view in the *q*_3_ direction is generally much smaller than in the other two directions. Therefore, it is necessary and essential to consider the field of view of the diffraction volume when implementing the algorithm. In order to limit the field of view, it is often more convenient to apply a constraint in real space rather than directly imposing it in reciprocal space, considering that the diffraction pattern is theoretically infinitely large. Since the Fourier transform of the corrected diffraction volume 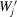 is an autocorrelation function, the envelope constraint with extent *D* in the *r*_3_ direction can be expressed as
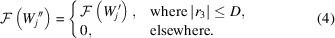
Considering the geometrical relation between real space and reciprocal space, for an arbitrary slice number *N*_*j*_ of the rebuilt diffraction grid, the parameters we need to adjust to make the field of view consistent with the desired (set) rocking angle range are the step size in the *q*_3_ direction, d*q*_3_, and the envelope *D.*

By using the preset angular step size dθ = 0.004° and the lattice parameter, we can derive a nominal 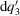 value of (2πdθ)/*d*, where dθ is given in radians. We found that the field of view was consistent with the desired rocking-curve range when 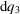 = 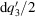 and *D* = 375 nm. The algorithm ran for 250 iterations to generate a diffraction intensity model *W* with *N*_*j*_ = 201 frames. An initial probability *P*_*jk*_ with a Gaussian distribution was set to build up our initial model according to equation (3). The continuity bias *n*_σ_ was fixed at 6, while the annealing coefficient β was initially 10^−5^ and then multiplied by 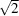 every 5 iterations. The algorithm was implemented and evaluated at MAX IV Laboratory computational cluster and took about 2.07 h (detailed in supplementary note 1 of the supporting information).

## 3. Results and discussion

### 3.1. Correcting simulated angular distortion

**Figure 2:**
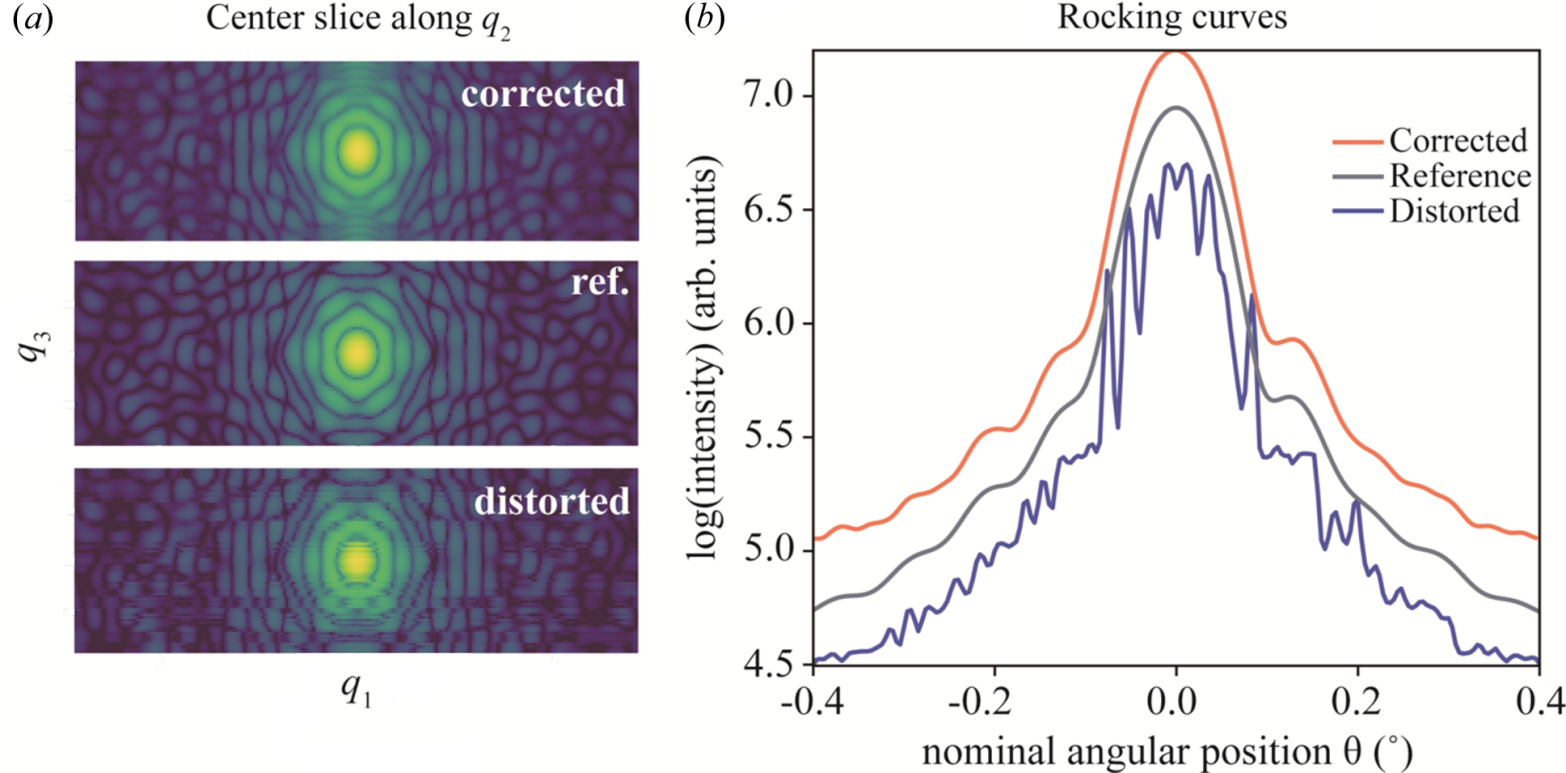
Bragg peak correction at Γ_θ_ = 2.81. (*a*) Center slice of the corrected, reference and distorted data. (*b*) Rocking curves (*i.e.* the total intensity) as a function of angle, in logarithmic scale, for the corrected, reference and distorted datasets. For clarity, the reference and corrected curves were vertically shifted by 0.25 and 0.5, respectively.

We tested the performance and limitations of the process for a range of Γ_θ_, but we first present the results for Γ_θ_ = 2.81 in some detail in Figs. 2 ▸ and 3 ▸. The center slices of the datasets along the *q*_2_ and *q*_3_ directions are displayed in Figs. 2 ▸(*a*) and 3 ▸(*c*), and the rocking curves of each dataset, as shown in Fig. 2 ▸(*b*), were calculated by summing the intensity of each frame along θ. The effect of the angular distortion can be clearly observed in both the diffraction pattern and the rocking curve in Fig. 2 ▸. In contrast, the corrected dataset is smooth and very similar to the reference in the high-intensity range between θ = [−0.3°, 0.3°] . For the low-intensity tails, some deviations can be observed.

**Figure 3:**
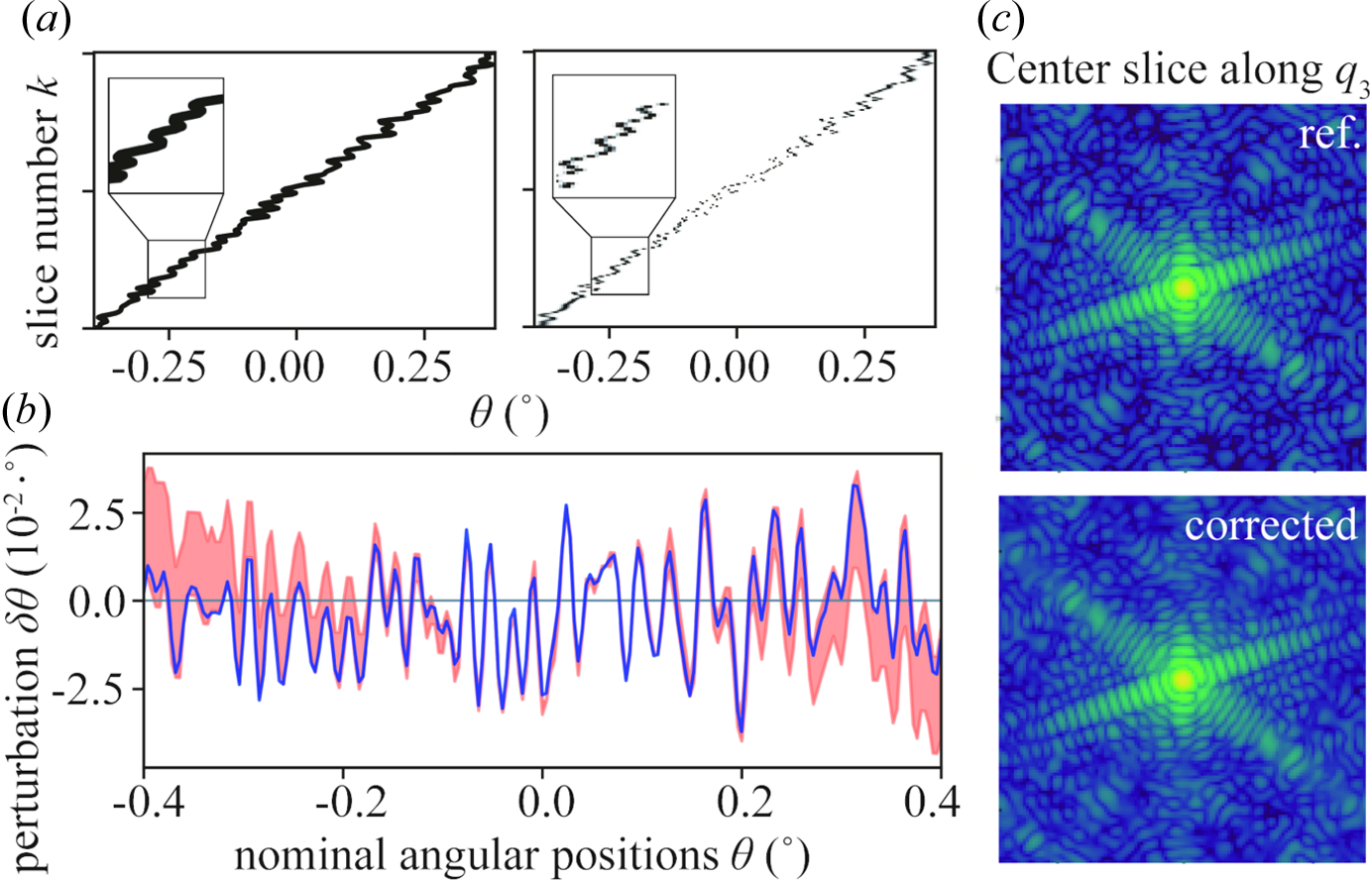
Rocking curves and angular positions at Γ_θ_ = 2.81. (*a*) Orientation trajectories. The left panel shows the input (distorted) trajectory, and the right panel shows the probability *P*_*jk*_ from the last iteration of the correction algorithm, which can be considered as the estimated trajectory θ(*k*). (*b*) δθ distribution (orange shaded area) and the ground truth (solid blue line). δθ is calculated from *P*_*jk*_. Since the probability is not a single value at each regular angular position, a threshold of 10^−3^ was applied here. (*c*) Center slices of the reference diffraction pattern and corrected diffraction volume *W* in logarithmic scale.

To make a more detailed comparison, we analyzed the distorted and the corrected angular trajectories. The normalized probability distribution *P*_*jk*_ calculated in the last iteration of the corrected algorithm gives the orientation trajectory for the rocking angle θ(*k*) as shown in Fig. 3 ▸(*a*), which recovers the input trajectory very well. The perturbation distribution δθ shown as the orange shaded area in Fig. 3 ▸(*b*) was obtained after thresholding the elements of the probability matrix *P*_*jk*_. The solid line represents the simulated preset δθ, which was calculated by subtracting the nominal trajectory from the input one. The preset δθ is well covered by the perturbation estimated by our method. One can observe that the distribution converges to a very small range in the middle part of the rocking positions and diverges to a larger range at both ends. This tendency is consistent with the deviation observed in the corrected rocking curve mentioned above. According to equations (1) and (2), *P*_*jk*_ is calculated from the cross correlation between the photon counts in each measurement *K*_*k*_ and the logarithm of the corrected diffraction volume *W*_*j*_ at each angular rocking position θ_*j*_. Both ends of the rocking curve have much lower photon counts (note the logarithmic scale), and it is therefore to be expected that the algorithm performs worse in these regions.

**Figure 4:**
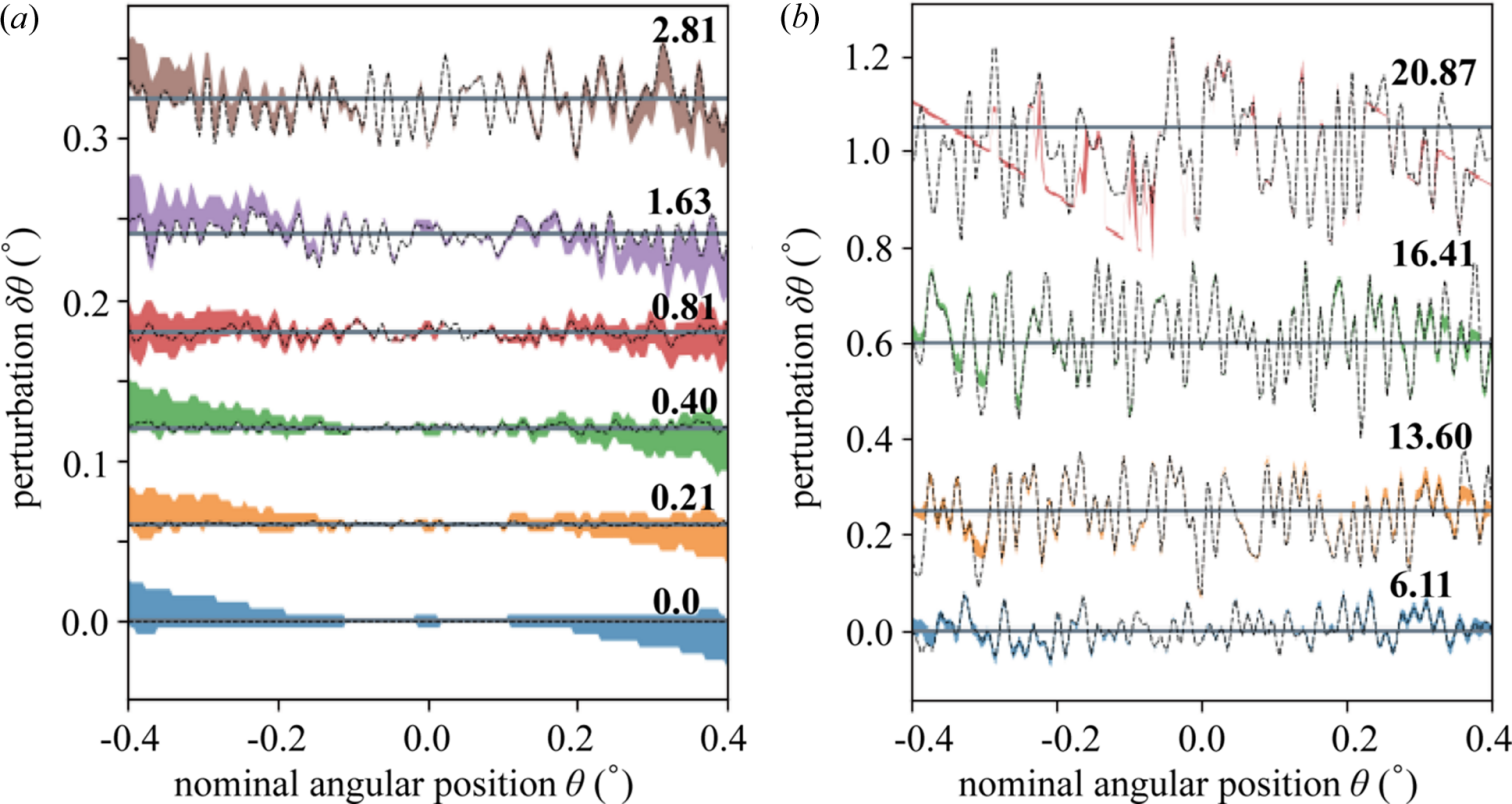
Recovered δθ for different Γ_θ_. (*a*) δθ for simulated datasets with different Γ_θ_ values starting from 0 up to 2.81. Distributions for each Γ_θ_ were shifted vertically for clarity. The black lines show the pre-defined δθ. Colored areas are the δθ calculated from the probability *P*_*jk*_ after applying a threshold value of 10^−3^. (*b*) δθ for simulated datasets with larger Γ_θ_ up to 20.87, as plotted in (*a*). The datasets were shifted vertically to separate the graphs for different Γ_θ_.

To investigate the robustness of our approach with respect to the δθ variation, more numerical examples with different Γ_θ_ were calculated. The Γ_θ_ value of these examples varied from 0 to 20.87. All these numerical examples were analyzed by the correction algorithm, with only minor tuning of d*q*_3_ and *D* to obtain optimal results. The estimated δθ returned from the correction algorithm and their ground truth δθ can be found in Fig. 4 ▸. The method works very well up to Γ_θ_ = 6.11, particularly in the central region with high photon counts. We notice that there seems to be a systematic linear error in the retrieved δθ values, especially for mild angular uncertainty (Γ_θ_ ≤ 2.81) cases. However, this is only observed outside of the central region. This behavior might come from the Gaussian bias, *n*_σ_, applied in the algorithm, or other high-order errors during the calculation of *P*_*jk*_. We also note that the field of view defined by *dq*_3_ and *D* was slightly wider than the ground truth in real space, which could result in a smaller angular range in θ.

For cases with even higher Γ_θ_, we find that the method can still give good results up to Γ_θ_ = 16.41 (which is around 0.066°, almost 10% of the total nominal rocking curve range). The estimated δθ covers the ground truth δθ very well in the range [−0.3°, 0.3°] of the angular rocking position θ_*j*_. However, the resulting δθ outside this range seems to be truncated by lines at both ends. The line-shape truncations of δθ, as shown in higher distortion case in Fig. 4 ▸, represent boundaries of the field of view defined by d*q*_3_ and the maximum *D*. The truncations observed in higher-distortion cases show the similar behavior as the linear trends observed in low- and mild-distortion cases discussed above. The datasets with higher Γ_θ_ might cover a larger angular rocking curve range than the nominal one, which means the angular position of some slices might be out of the field of view. We performed a test with Poisson noise distribution (see Fig. S2 of the supporting information) and found that the algorithm performed similarly well in this case.

The highest level of distortion that we attempted, Γ_θ_ = 20.87, is shown in Fig. 4 ▸(*b*). In this case, the trajectory θ(*k*) (see Fig. S1) can be described as random. As shown by the previous results, the algorithm has very good performance in the central region of the rocking curve range, and it was forced to work in a much narrower field of view by setting a smaller *D*. It is rather surprising that the process could still give us a reliable result, albeit in a limited angular range. Thus, we find that the maximum average perturbation that can be reconstructed is about 0.08°, which is similar to the fringe spacing. Note that such a large δθ is substantial in comparison with the overall angular range (Δθ = ± 0.4°).

### 3.2. Reconstructing of continuous angular scans

Traditional BCDI relies on step scanning, where the sample is rotated between frames and is stationary during the acquisition. Step scanning leads to time lost as overhead when the motors start, move and stop, as well as in the control software, and with higher coherent fluxes and shorter counting times this becomes a comparatively larger problem. The stop-and-go motion of the motor can also lead to mechanical instabilities, especially with heavy setups for *in situ* experiments. Alternatively, the rotation motor can be scanned continuously in a so-called fly scan mode (Li *et al.*, 2020 ▸), which has become a common approach for real space scans in ptychography (Clark *et al.*, 2014 ▸; Pelz *et al.*, 2014 ▸).

**Figure 5:**
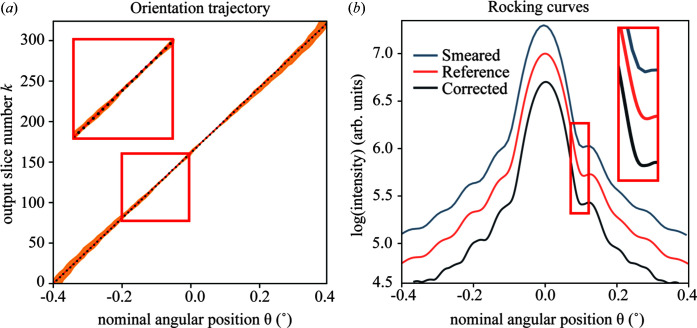
Continuous scan simulation with no distortion. In continuous scanning, the rotation motor moves during acquisition, which leads to smearing. (*a*) Orientation trajectories for reference (red solid line) and smeared (black dot) datasets. The shaded area represents the possible angular position is calculated from *P*_*jk*_ by applying a threshold of 10^−4^. (*b*) Rocking curves in logarithmic scale for the smeared, reference and corrected datasets. For clarity, the reference and smeared curves were vertically shifted by 0.25 and 0.5, respectively.

In the continuous scan, each diffraction pattern represents an average angle during a single exposure time, which is a form of smearing in reciprocal space that could potentially lead to a loss of resolution. We investigated whether the algorithm could reconstruct the movement during a continuous scan, as shown in Figs. 5 ▸ and S3. In our simulation, we modeled this smearing effect by averaging four adjacent angular positions in a single pattern, reducing the 324 frames in the reference to 84. We defined the reconstruction such that it also has 324 frames, aligning with the frame count of the reference data, but note that this is not necessary.

The result of the reconstruction is shown as a trajectory in Fig. 5 ▸(*a*) and as a rocking curve in Fig. 5 ▸(*b*). We find that the original angles can be recovered very well. The minor ‘peak’ observed at the first fringe in the rocking curve of the smeared data, highlighted by the red boxes in Fig. 5 ▸(*b*), signifies a reduction in resolution attributable to the smearing effect happening in the continuous scan measurement. The comparison of the rocking curves among the reference, smeared and corrected data demonstrates the capability of the algorithm to effectively reconstruct the data from a dataset with lower resolution. However, the slight wiggles at the ends of the rocking curve of the corrected data show that it does not quite match the high resolution of the reference data. This is also noticeable in the wider range of angles shown by the shaded area in Fig. 5 ▸(*a*), in line with the results for the distorted scans above.

We also simulated a combination of distortion and continuous scanning, where the random distortion was applied before the smearing. The reference, distorted and corrected datasets consist of 164 frames each, whereas the smeared dataset contains 41 frames. Although the smearing reduces the distortion effects to some extent, our approach still demonstrates superior performance, as shown in Fig. S3.

In this preliminary investigation, we described the smearing effect in a continuous scan by simply averaging four adjacent angular positions. This situation does not accurately match the assumptions for equations (1) and (3), but nevertheless we obtained satisfying results. We also note that the particles in the original paper by Björling *et al.* (2020 ▸) were rotated continuously. This suggests that *P*_*jk*_ is a reasonable representation of the continuous scanning scenario. A more accurate theoretical model could lead to better results, but this is outside the scope of this work.

### 3.3. Phase retrieval of the simulated data

The correction is applied as a pre-processing algorithm, performed before and independently of the phase retrieval. We have so far discussed the results in reciprocal space, where the correction is easier to analyze. However, we also investigated the impact on the phase-retrieved objects, since this is the idea of a BCDI experiment. The plotted rocking curve and the estimated δθ shown above indicate that the method works very well within the angular region [−0.3°, 0.3°]. The mismatch part outside this portion might lead to some unexpected results after phase retrieval (see Fig. S4). Therefore, the low-intensity parts at both ends of the rocking curve were cropped in the resulting corrected diffraction dataset *W* from the algorithm as shown in Fig. 2 ▸(*a*). The cropped-corrected diffraction volume *W* was then processed using the *PyNx* software for phase retrieval (Favre-Nicolin *et al.*, 2020 ▸).

**Figure 6:**
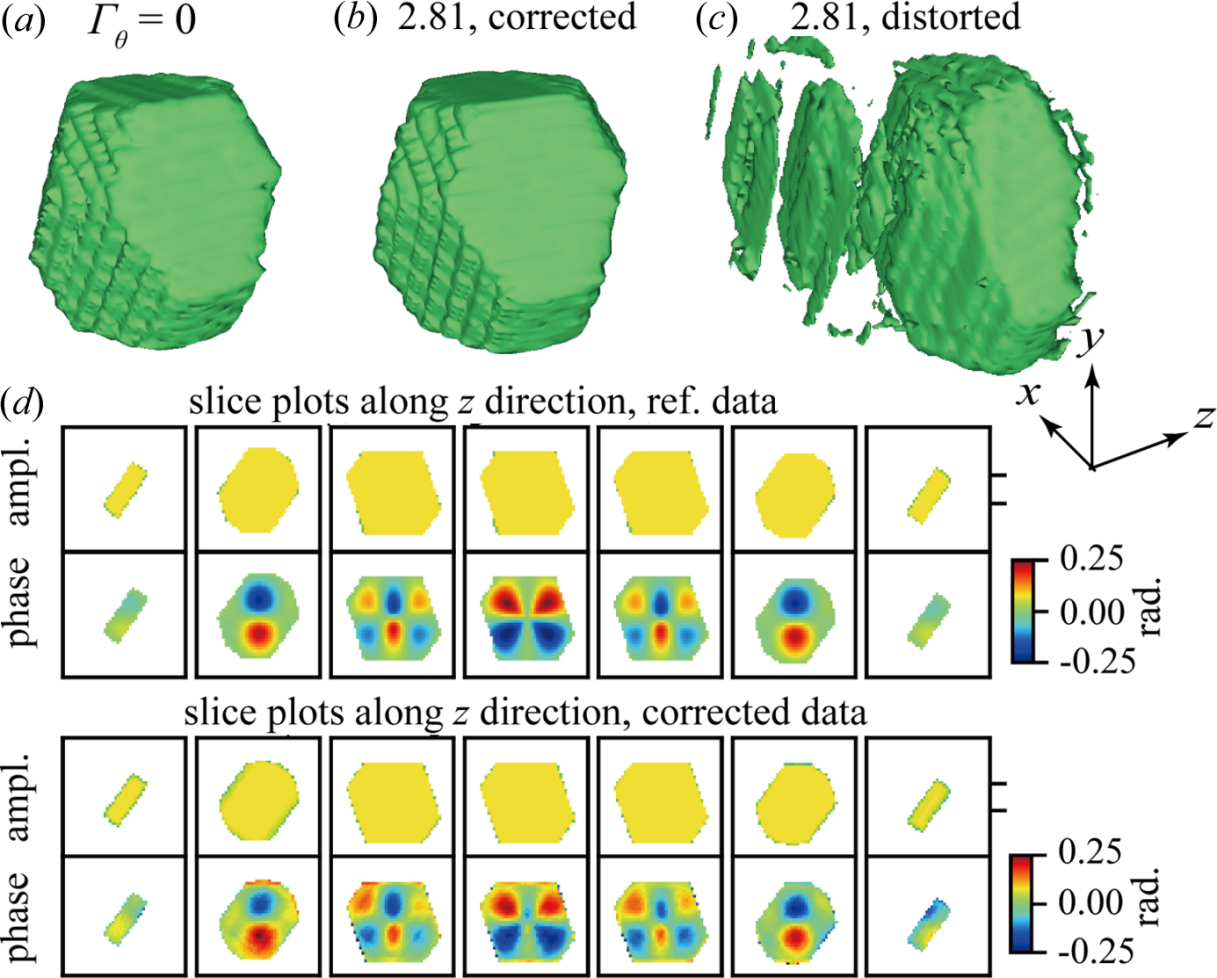
Effect of angular correction on phase retrieval for Γ_θ_ = 2.81. Morphology of the particle reconstructed from the (*a*) Γ_θ_ = 0 dataset, (*b*) corrected dataset and (*c*) input dataset via phase retrieval, separately. Identical phase-retrieval parameters were applied. (*d*) Internal phase distribution recovered by phase retrieval shown as slice plots. The slice plots are along the *z* direction in real space. The upper panel shows the internal phase distribution of the reference; the lower panel shows the reconstructed internal phase distribution of the corrected dataset *W* at the same positions as in the upper panel.

The reconstruction from the distorted datasets with Γ_θ_ = 2.81 is shown in Fig. 6 ▸(*c*). We used the default settings as much as possible, avoiding fine-tuning in order to focus on the effects of the distortion. The default reconstruction process of *PyNx* consisted of 600 relaxed averaged alternating reflection (Luke, 2005 ▸) iterations followed by 200 error-reduction (Gerchberg, 1972 ▸; Fienup, 1982 ▸) cycles and a shrink–warp (Marchesini *et al.*, 2003 ▸) support threshold with an amplitude coefficient of 0.1–0.5. The initial support was obtained based on the autocorrelation of the input 3D diffraction data. In total, 1000 reconstructions were carried out and only 20 of them with the highest free log-likelihood values were selected and combined for the final result (Favre-Nicolin *et al.*, 2020 ▸). The reconstructed morphology from other phase-retrieval algorithm, such as hybrid input–output, are shown in Figs. S5(*d*)–S5(*f*), together with their phase-retrieval transfer functions.

The distorted data with Γ_θ_ = 2.81 results in a poor reconstruction with multiple sidelobes. Comparably poor results are observed with other phase-retrieval algorithms, and we observed similar sidelobes for other high distortions (not shown). In the phase-retrieval process, sidelobes might appear when a large support is applied; these can be minized using a more compact support with fine-tunning, as done in the work by Calvo-Almazan *et al.* (2019 ▸). However, we would like to point out that the example shown in this section (Γ_θ_ = 2.81, 281% of the step size) exceeds the capability of the phase-retrieval based method. In comparison, the reconstruction from *W* is very similar to the reference. Both the morphology of the particles reconstructed from the reference and *W* demonstrate the truncated-octahedral envelope very well. The variation of the reconstructed phase distribution in the *z* direction is also consistent with that of the reference. The more accurate morphology and phase distribution obtained from the corrected data demonstrate the effectiveness of our algorithm in correcting artifacts in phase-retrieval reconstruction introduced by angular distortion in the rocking direction.

**Figure 7:**
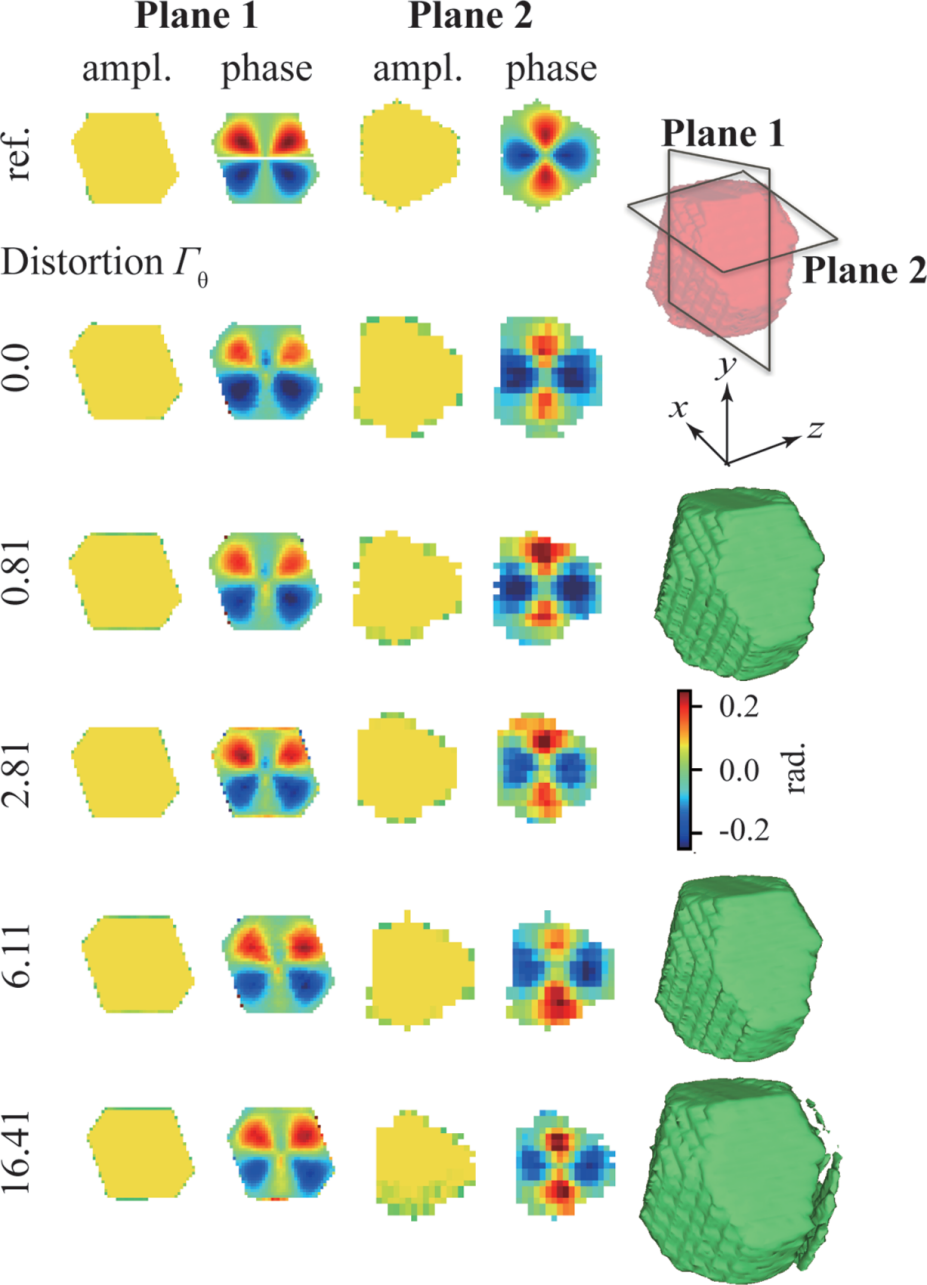
Phase-retrieved objects for different levels of Γ_θ_. Note that the reference object here is the model, before the simulation of diffraction and phase retrieval. Planes 1 and 2 represent two perpendicular sections of the nanoparticle, as illustrated in the figure. The first and the third columns show the amplitude of the reference particle and reconstructed particles from different distorted datasets, respectively. The second and the fourth columns display the corresponding phase distribution. Identical phase-retrieval parameters were applied for all reconstructions. The last column displays the reconstructed morphologies from various levels of Γ_θ_.

The impact of different Γ_θ_ on the phase retrieval is presented in Fig. 7 ▸. A cross-section slightly above the midpoint along the *y* direction was chosen to demonstrate the variation of the phase distribution along the *z* direction. The amplitude and phase plots of the reference dataset are taken directly from the simulation, whereas those of the corrected datasets are the reconstructions after phase retrieval. The blurring of plots can be attributed to the default Gaussian convolution kernel applied in each relaxed averaged alternating reflection iteration according to Favre-Nicolin *et al.* (2020 ▸). Together with the results shown earlier in Figs. 6 ▸(*a*) and 6 ▸(*b*), the morphology reconstruction from the corrected datasets *W* with different Γ_θ_ (see Fig. 7 ▸) is robust. Although there are some sidelobes appearing in the reconstruction at Γ_θ_ = 16.41, the main features of a truncated octahedron can still be clearly observed. The phase distributions are challenging to compare since even a minimal real space shift during the phasing process makes the slices look quite different. The reconstructed phase distribution seems consistent under the variance of δθ. From very subtle to large angular distortion of up to Γ_θ_ = 16.41, the algorithm has a robust performance not only for the reconstruction of the morphology but also of the phase. Thus, we find that the improvements observed in reciprocal space result in much better phase-retrieved objects.

### 3.4. Algorithm on real experiment data

**Figure 8:**
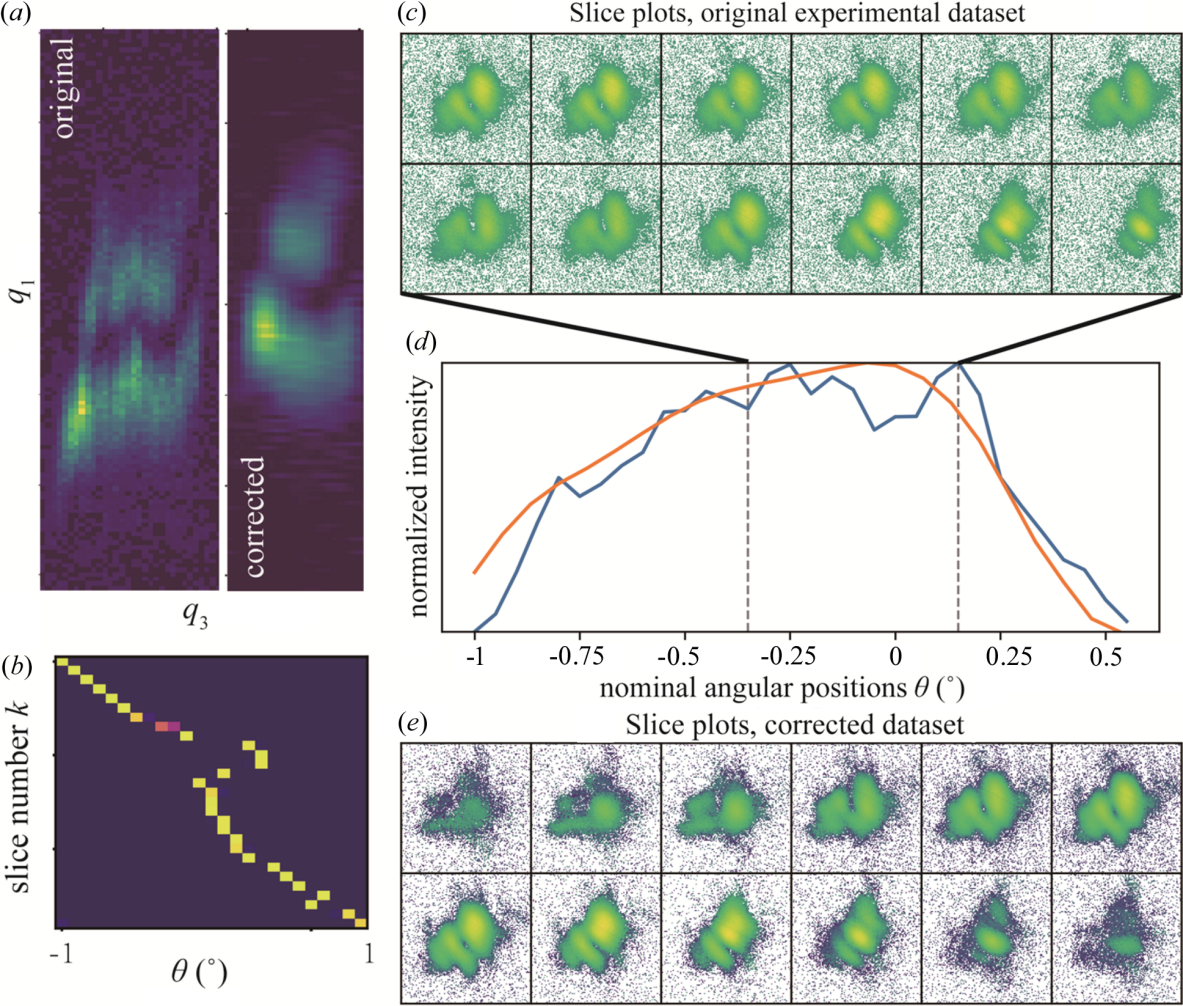
Correcting experimental data. (*a*) Central slice along the *q*_2_ direction of the real experimental dataset (left panel) and the corrected dataset *W* (right panel). (*b*) Trajectory θ(*k*) (probability metric *P*_*jk*_) obtained from the last iteration of our correction algorithm. (*c*) Measured diffraction frames (logarithmic scale) in the region highlighted by the gray dashed lines in the rocking curve. (*d*) The last frame indicates that the measurement was also affected by the roll angle ω, which was aligned by the center of mass in the following step. (*d*) Normalized rocking curves of the real experimental data (blue line) and the corrected diffraction volume *W* (orange line) in logarithmic scale. (*e*) Slice plots of the corrected volume *W* in logarithmic scale. It corresponds to the orange line shown in (*d*). A threshold of 2 was applied to the corrected volume *W*.

Finally, we apply our method to real experimental data. We observed angular drift in a BCDI experiment imaging a 200 nm Ge nanoparticle on the P10 beamline at PETRA III (details are given in supplementary note 3 of the supporting information). The Bragg peak of the measured Ge particle was supposed to be evenly sampled 50 times over a rocking angle range of ±1°. However, as shown in Figs. 8 ▸(*a*), 8 ▸(*c*) and 8 ▸(*d*), the diffraction patterns vary non-systematically, forward and backward during the scanning.

Prior to sending the experimental data to the correction algorithm, several pre-processing steps were performed (see supplementary note 2 of the supporting information). The whole dataset is based on the calculated center of mass of the brightest frame to compensate for the influence caused by the roll angle ω. This step assumes a high degree of symmetry in the nanoparticle, and a future improvement could be to include the roll angle in the correction alogorithm. d*q*_3_ was estimated from the first minimum of the brightest frame, and the maximal *D* was set to 200 nm. The other parameters were the same as described above. After 80 iterations, the algorithm generated a corrected diffraction volume *W* with 25 frames taken from the experimental data as input. Figs. 8 ▸(*a*), 8 ▸(*d*) and 8 ▸(*e*) show the results. Compared with the original experimental data, the central slice of the corrected dataset *W* and its rocking curve are much smoother. The main features of the Bragg peak were rebuilt well by the procedure, as shown in Fig. 8 ▸(*e*). Fig. 8 ▸(*b*) demonstrates the trajectory θ(*k*) obtained from the last iteration. The trajectory is consistent with the observed angular drift that appeared during the rocking curve scanning. We note that the deviation from the nominal angle is the largest near the peak of the rocking curve, at the Bragg condition. Unfortunately, we could not obtain a reasonable phase-retrieval reconstruction even from the corrected frames due to the poor quality of the original experimental data.

## 4. Conclusions

We devised a pre-processing algorithm for correcting angular distortions in BCDI measurements on both simulated and real experimental data. The process returns a reliable estimate of the orientation trajectory θ(*k*) for Γ_θ_ of up to 16.41× the step size dθ = 0.004°. We find that this limit is relatively sharp. Naturally, one can expect a large variation depending on, for example, sample features, counts and number of frames, but it is not practical to investigate all these degrees of freedom here. The reconstruction obtained from the corrected diffraction volume *W* via phase retrieval could clearly display the main features of the phase distribution and the morphology of the simulated particle. We also found that the algorithm could correct severely distorted experimental data. Since this is a pre-processing method, the user still has full freedom in the phase-retrieval step.

The obvious purpose of the algorithm is to make it possible to use experimental data that would otherwise be discarded. However, it could also be used on data that appear to be of high quality. Modest angular distortions could be difficult to recognize in an experiment, where there is no ground truth. In these cases, pre-processing could result in higher-quality phase retrieval. We have also shown that our approach could be useful in continuous-scanning BCDI, which is expected to become more popular with increased coherent fluxes and shorter acquisition times. Another use could be as a quick quality control of experimental data. The reciprocal space analysis could provide faster and more direct insight into potential problems with angular distortions during acquisitions, compared with analysis of phase-retrieved objects. This opens the possibility for using BCDI under more challenging conditions with smaller and less attached particles, and to fully exploit the intensity of beams from fourth-generation synchrotrons.

## Supplementary Material

Supplementary notes and figures. DOI: 10.1107/S1600577524006507/gy5065sup1.pdf
