# Supplementary material for: Correcting angular distortions in Bragg coherent X-ray diffraction imaging
Source: J Synchrotron Radiat. 2024 Aug 8;31(Pt 5):1308–16. doi: 10.1107/S1600577524006507 (PMC11371051; doi:10.1107/S1600577524006507)
Supplement: Supplementary file 1 [file s-31-01308-sup1.pdf]

JOURNAL OF
SYNCHROTRON
RADIATION

Volume 31 (2024)

Supporting information for article:

Correcting angular distortions in Bragg coherent X-ray diffraction imaging

Huaiyu Chen, Dmitry Dzhigaev, Alexander Björling, Fabian Westermeier, Mikhail Lyubomirskiy, Michael Stuckelberger and Jesper Wallentin

Supplementary Notes

Supplementary Note 1 Computational time

A dataset with $\Gamma_\theta = 2.81$ was used to evaluate the processing time, with identical parameters as stated in the Details of the algorithm and simulation section above. The algorithm was run on the HPC server at MAX IV, using one CPU node and 20 hyperthreads. The total processing time was approximately 2.07 hours.

Supplementary Note 2 Pre-processing operations to the real experimental dataset

The size of the original frames recorded by the detector is 2167 × 2070 pixels. We cropped the frames to a size of 200 × 200 pixels at the locations where the Bragg peak was recorded in the original frames. Since the dataset had a very noisy background, we applied a threshold of 35 counts to slightly improve the quality of the dataset.

Supplementary Note 3 Experimental data obtained at PETRA III, DESY.

The experiment aimed to perform BCDI on a single Ge nanoparticle with a grain size of 200 nm. The goal was to obtain the strain distribution within the nanoparticle. A monochromatic X-ray beam with an energy of 13.088 keV, focused to achieve a Full Width at Half Maximum (FWHM) of approximately 2 μm, was used. The relative spread in wavelength was around 10^{-4}. The setup included an Eiger X4M detector with 2016 × 2070 pixels, positioned at detector angles $(\gamma, \delta) = (9.42^\circ, 11.77^\circ)$, 1.83 m downstream from the sample. To satisfy the Bragg condition, the sample was rotated to $\theta = 11.16^\circ$. The 3D diffraction patterns of the Ge nanoparticle were obtained by slicing it into 50 frames over a relatively angular range of [-1°, 1°]. Signal enhancement was achieved by integrating 40 exposures per frame at each angular position during the rocking curve measurements.

Supplementary Note 4 Our code for simulations and algorithms, which form the core of analyses presented herein, are available for review and replication. The real dataset used for validation has also been made publicly accessible. All material can be found on our GitHub repository at https://github.com/chhy0426/Angular-correction-algorithm-for-BCDI

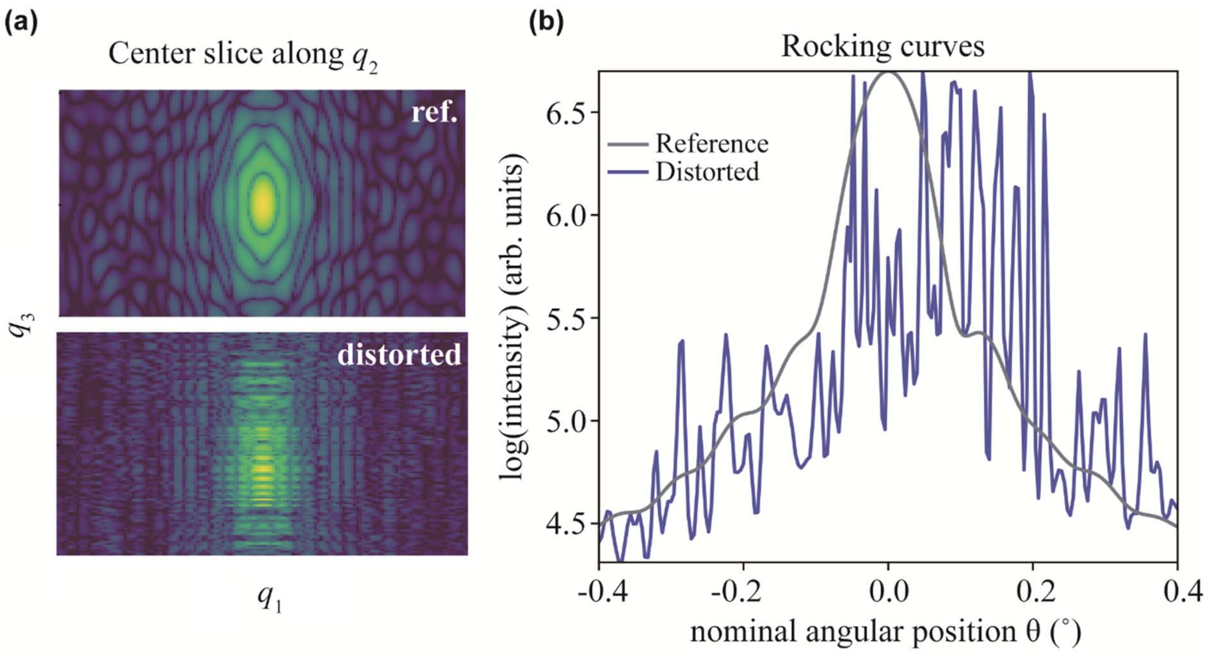

Figure S1 $\Gamma_\theta = 20.87$, center slice along q_2 for the reference, and the distorted dataset, as well as their rocking curves. (a) the center slice of the reference and the distorted dataset. The top panel shows the simulated data without any angular distortion on rocking angle θ. The bottom panel represents the input data to the algorithm, simulated with $\Gamma_\theta = 20.87$. (b) rocking curves in logarithmic scale for the reference and the distorted datasets.

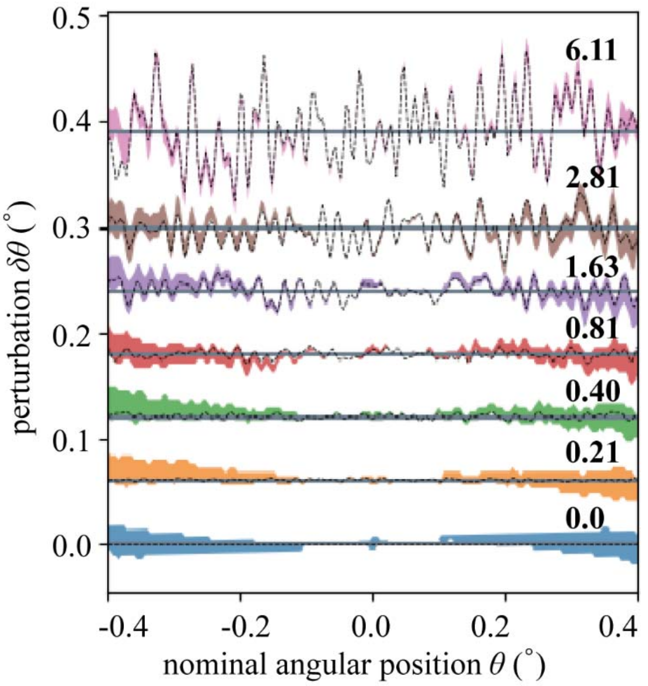

Figure S2 Perturbation distributions $\delta\theta$ with Poisson noise. Perturbation distributions $\delta\theta$ for noisy simulated datasets with different distortion Γ_θ up to 6.11. The distributions for each distortion Γ_θ are shifted vertically for clarity. The black lines show the pre-defined angular perturbations $\delta\theta$. Colored areas are the perturbation distributions $\delta\theta$ calculated from the probability P_{jk} after applying a threshold value of 10^{-3}.

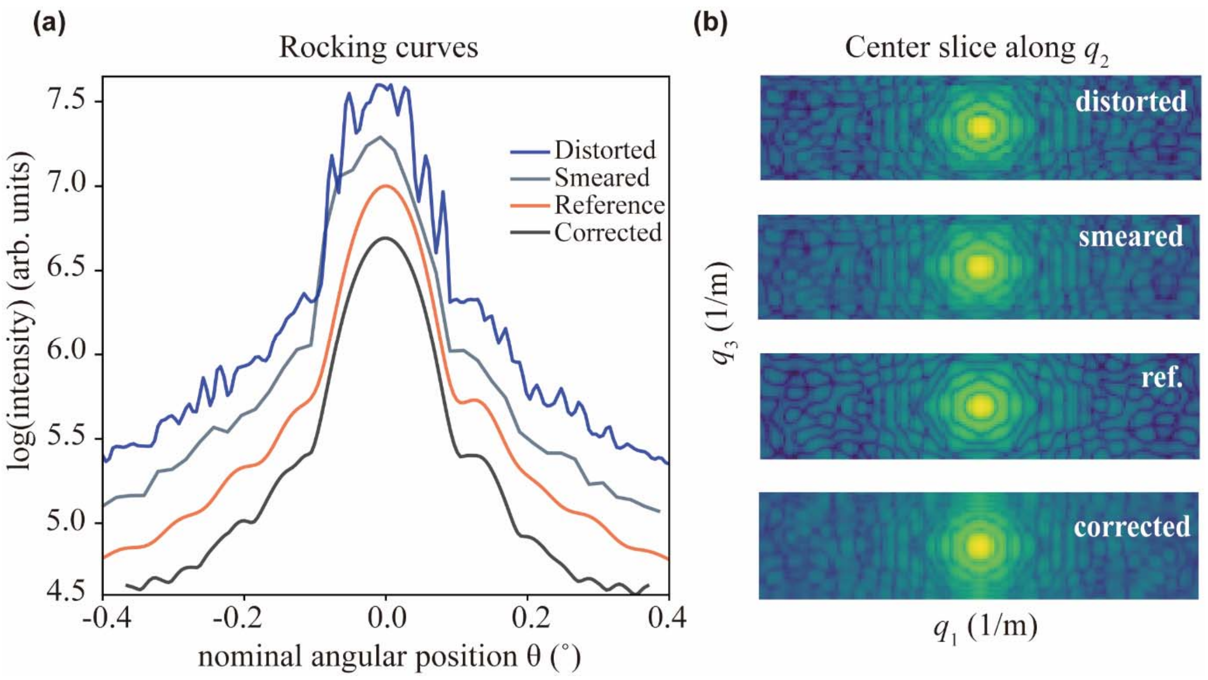

Figure S3 Reconstruction of a continuous scan with distortion. The distortion ($\Gamma_\theta = 2.35$) was applied before the smearing. (a) rocking curves, i.e., the total intensity as a function of angle, in logarithmic scale, for the distorted, smeared, reference and corrected datasets. For clarity, the reference, smeared, and distorted curves were vertically shifted by 0.25, 0.5 and 0.75, respectively. The reference distorted and corrected dataset have 164 frames, while the smeared dataset only has 41 frames. We simulated the smearing effect with respect to the distorted data and used the smeared dataset as the input of the algorithm. (b) the center slice of the distorted, smeared, reference and corrected datasets.

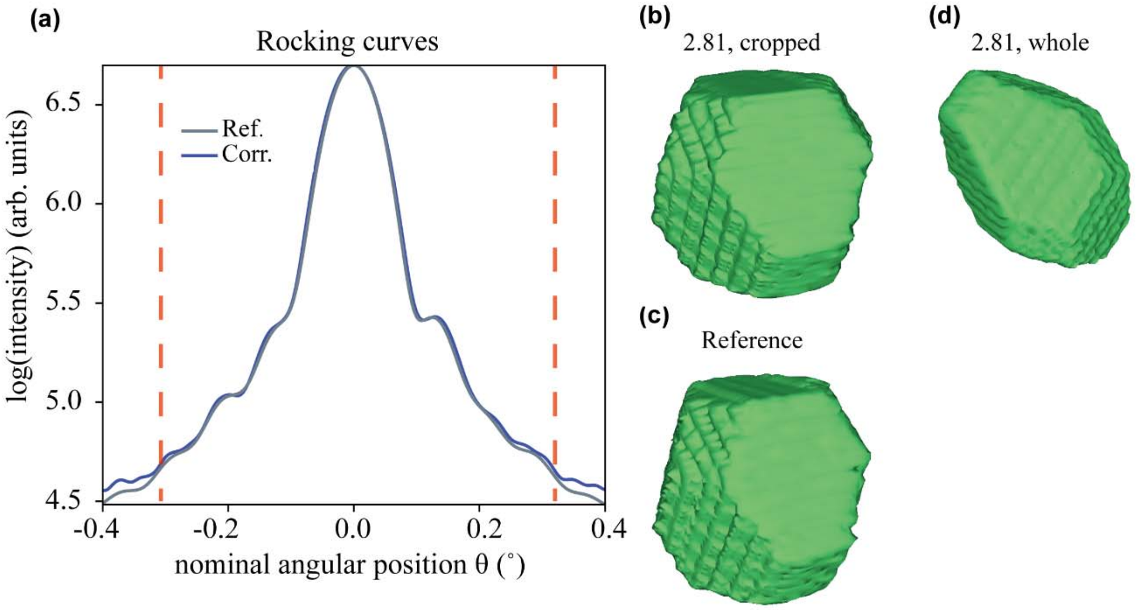

Figure S4 Illustration of cropping. (a) Rocking curves of reference and corrected dataset with $\Gamma_\theta =$ 2.81. The region enclosed by red dash lines is the angular region with high photon counts. (b) The reconstructed morphology from the cropped dataset as illustrated in (a). (c) The reconstructed morphology from the reference data. (d) The reconstructed morphology from the corrected dataset. The mismatch part shown in the rocking curves seems to lead to artifacts in the reconstruction. The features of a truncated octahedron can still be clearly observed in (d). However, the orientation and the length in each direction are different than the reconstructions shown in (a) and (c). The reconstructions from the reference and cropped corrected dataset are very similar.

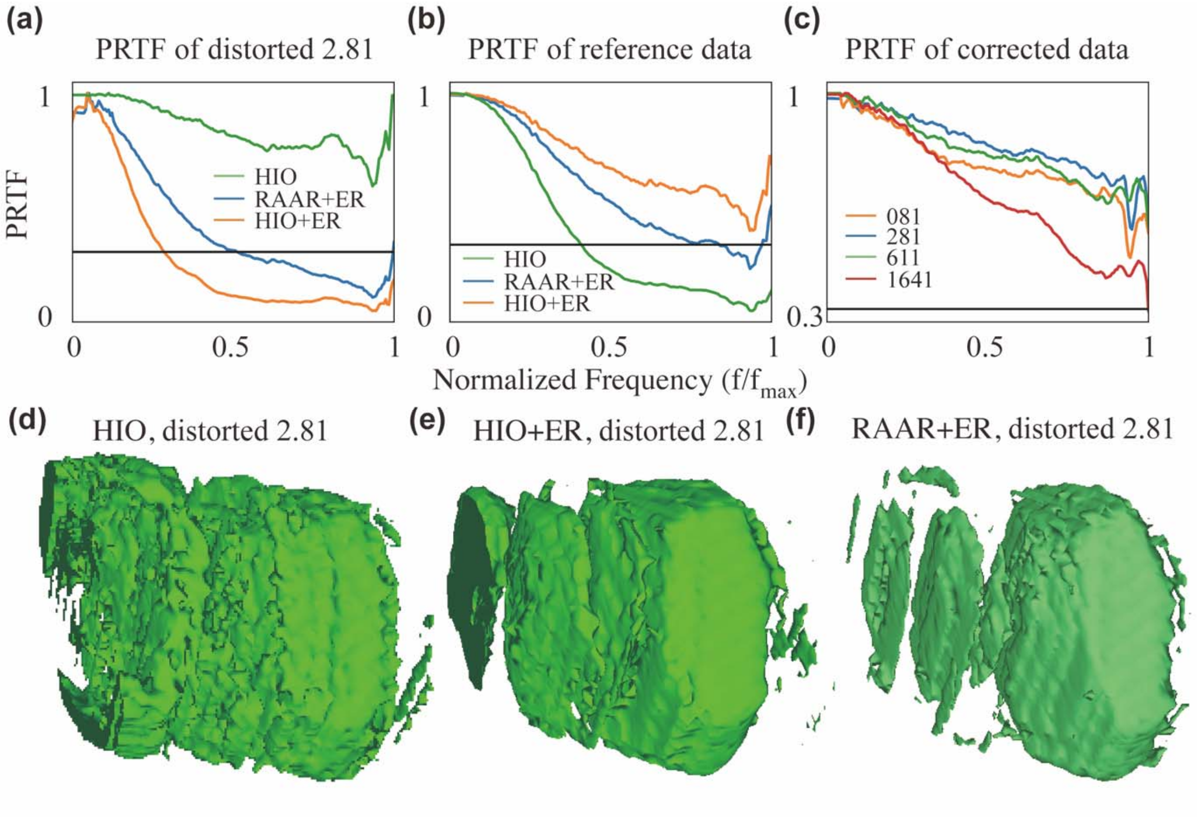

Figure S5 Evaluation of phase retrieval. (a) – (b) Phase Retrieval Transfer Function (PRTF) of the reconstruction from the distorted dataset $\Gamma_\theta = 2.81$ and the reference data $\Gamma_\theta = 0$ via RAAR + ER, HIO + ER and HIO. RAAR + ER is the default phase retrieval algorithm in PyNx software. All three phase retrieval algorithms used the same setting in PyNx. (c) Phase Retrieval Transfer Function (PRTF) of the reconstruction from the corrected datasets showed in Figure 7. All reconstruction in (c) employed the RAAR+ER algorithm. The black solid line in (a) – (c) represent the value of e. (d) – (f) Morphology reconstructions from the distorted dataset $\Gamma_\theta = 2.81$ via RAAR + ER, HIO + ER and HIO. These correspond to the PRTF curves in (a).
